# Case and Dissection of Aneurism of the Aorta, in Which There Had Been Very Slight Irregularity of the Pulse, with Many Other Remarkable Appearances, Not Suspected during Life

**Published:** 1815-04

**Authors:** John Haxby

**Affiliations:** Pontefract


*For the Medical and Physical Journal.*

### *Case and Dissection of Aneurism of the Aorta, in which there had been very slight Irregularity of the Pulse, with many other remarkable Appearances, not suspected during Life;* by JOHN HAXBY, of Pontefract, M.D.: *containing also Observations,* by Dr. BAILLIE, of London.

LIEUT.-COL. T., æt. 39, endowed with an excellent constitution, entered into the army at the age of 16, and indulged in all the convivialities of the table without restraint. While in the West Indies, he suffered from the fever of the climate, and had the jaundice severely. In a short time he regained his health, and continued free from indisposition indisposition till within a year and a half, or two years, of his decease; at which period he became subject to dyspeptic complaints, gradually lost his natural vivacity and equanimity; the complexional bloom of health, and plumpness of habit, being exchanged for sallowness and emaciation. The faculty, whom he at the time consulted, treated the complaint as a diseased liver, and administered mercurial medicines freely, with apparent advantage to his general health.

The Col. first consulted me in the month of April last, complaining that his sleep was much disturbed, that his appetite failed him, and that he had frequent palpitations at his heart on any increased exertion. His complexion and habit were as above described; his pulse somewhat quicker and more dilated than natural, with occasional intermissions at long intervals. I was not without my suspicion that there was a tendency to aneurism of the heart or its larger vessels, which, if it did not terminate fatally by rupture, might end in hydrothorax. I prescribed a slight tonic, combined with diuretics, and recommended a continuation of the pilula hydrargyri, which he had been in the habit of taking for the supposed affection of the liver. I was agreeably surprised with the result of this treatment, which very materially relieved all the symptoms, and gave encouragement to hope that he might progressively regain his former vigour of health. Unfortunately, however, about this period, he took cold, from incautious exposure to the easterly winds, then very prevalent. The family apothecary had the exclusive management of him for three weeks, when I was desired to visit him at his own house, a distance of three miles from home, which hitherto he had preferred riding, from a persuasion that the exercise was conducive to his recovery.

I found him labouring under all the symptoms of atonic croup. The fauces internæ were exceedingly relaxed and pallid; the voice was gone, and inspiration was performed in a slow protracted manner, as if the air had to be drawn through a tube of contracted diameter. Every attempt to cough was followed by great anxiety and exhaustion, without any expectoration, except of viscid saliva. The pulse was small, obstructed, and slow; not exceeding 60, and sometimes not attaining even to 50 beats in a minute. As there was no thirst or any other symptom of fever, I considered the affection to be a species of chronic croup, and recommended astringent gargles, a blister to the throat, and expectorating medicines of ammoniacum and squill; particularly enjoining stillness, and an uniformly warm temperature. No amendment ensued from this plan, which was persisted in for some days, with occasional slight variation in the the expectorants. I then ordered the vapour of hot water medicated with vinegar, æther, &c. to be inhaled as often and as long at a time as was found convenient or beneficial; and the throat to be rubbed with mercurial ointment. All proved unavailing. The sleep of my patient now became disturbed, and the recumbent posture, which before had been most favourable to his tranquillity, proved a source of uneasiness. He found great difficulty in undressing himself, in consequence of increased dyspnœa, especially when his arms were extended, and his body inclined forwards. He was obliged to use the greatest deliberation in walking up stairs, and the anxiety he felt during a paroxysm of coughing was excessive. No expectoration followed, though, from the peculiar sound at the time of coughing, there was reason to apprehend that some viscid lymph was extended across the trachea, which, from the violent expulsory effort, must every moment be detached and discharged.

An eminent and experienced physician, Dr. Walker of Leeds, was called in consultation. He coincided with me in the propriety of continuing the local treatment of blistering the fauces, inhaling medicated vapour, and gargling with astringent and stimulating gargles. He advised also an emetic, which, from a fear of suffocation, I had hitherto forborne to recommend. Purgatives too formed part of the plan of treatment, from a belief that the liver, which gave pain on pressure, was a principal seat of disease; though I never heard my patient make any complaint of this organ previously to the examination by Dr. W. The compound liniment of quicksilver was rubbed in on the right side, and the pilula hydrargyri exchanged for the submuriate of mercury. He bore the operation of the emetic very well, and thought himself rather relieved by it. In consequence of the bowels being too much affected by the submuriate of mercury, the original pill was resumed, and, together with the liniment, was persisted in for a great length of time, without any ptyalism or unpleasant consequence supervening. The season being now agreeably warm, our patient was frequently indulged with an airing. No material advantage resulted from our combined efforts, except that the voice became gradually more audible. The emetic was repeated, but occasioned disappointment, requiring an increase of the dose, which frequently produced only one feeble effort of vomiting, while the bowels were much harassed with its secondary operation. The pulse now became unequal in its rate, beating sometimes 60, at other times 80 or 90, strokes in a minute, but always being slowest when the respiration was most difficult, and especially during the paroxysm of coughing.

 I described

I described the case to Dr. Baillie of London, who gave no encouragement to our hopes, considering the symptoms as indicative of some disease in the inner surface of the wind-pipe, with, probably, a disease of the liver, and a tendency to hydrothorax. He ordered four or five leeches to be applied twice a-week to the upper part of the sternum, if his strength would bear the application so often; a combination of the pilula hydrarg. with digitalis and squill, night and morning; and a slight expectorant of gum ammoniac dissolved in camphorated mixture, with the addition of a little spirit of æther, twice during the day.

The air of Thorp-Arch being esteemed remarkably fine, Col. T. took up his abode there for a fortnight; at the expiration of which period he returned home disappointed and dejected, more pallid and emaciated. He had, of late, experienced more pain and discharge than usual from the hæmorrhoids, to which he had been subject for some years, and could not, in addition, bear the small evacuation caused by the leeches.

A medical friend of the patient's encouraged him with the relation of a case, apparently similar to his own, which, after six weeks successive blistering of the throat, was completely cured by the expulsion of a viscid membranous lining of the trachea. I had no expectation of a result so favourable, being convinced, from the tone of my patient's voice, which, when exerted, was perfectly natural, that no such morbid lining existed; yet I acceded to the proposal of a succession of blisters. I should, however, have preferred a seton passed on each side of the trachea. The event justified my apprehension, and dispirited the patient.

The officers of his regiment having assembled at York, the Col. thought it necessary to give them the meeting. He was so exhausted by the exertion of talking to them, that he was obliged to have recourse to Dr. Best for assistance in the night, being threatened with suffocation. He was relieved from this alarming state by an emetic; and was, by experience, convinced that the original plan of mild expectorants combined with the digitalis and the pilula hydrargyri was ultimately most to be depended on. Still, however, no progress was made towards recovery, and the Col.'s patience, as well as that of his friends, began to be exhausted. Dr. Crowther, of Wakefield, was called in. He agreed with me as to the probable nature of the complaint being a thickened state of the internal membrane of the trachea, and advised repeated vesication of the part, with the subsequent application of the ceratum sabinæ to promote the discharge. He also proposed a solution of assafœtida. This, not proving

 efficacious, efficacious, was soon discontinued. The blistering process, having been so long ineffectually submitted to, was now objected to by the Col. who consented to have a seton inserted, as I had before proposed; this, however, from his becoming worse about this period, was deferred, and never afterwards put in practice.

He found great difficulty, about this time, in lying on the right side, from a sense of suffocation. On examining the chest in this posture of the body, I found that the heart did not beat in its usual place, being protruded more towards the centre of the sternum; nor did its pulsations quite correspond with those of the radial artery; the aorta descendens, as well as ascendens, with its immediate branches, beating more strongly than might be expected from the generally debilitated state of the patient, and the slenderness of the pulse at the wrist, especially in the left arm. In making this examination, I was, for the first time, struck with a peculiarity in the breathing, which I heard distinctly on applying my ear to the thorax, and even at a greater distance, and which the patient had himself before remarked. After the air had been drawn in by a protracted and laborious inspiration, and was about to be returned, a peculiar sound was heard, as if the air had regurgitated, and passed rumbling along into a large and deep cavern.

Having had an interview with Dr. Storer of Nottingham, I communicated the particulars of the case to him. He advised dry cupping of the part, electricity, and the nitras argenti in large doses. Electricity had been before suggested by a medical friend of the patient, and had been tried, without advantage. The nitras argenti did not agree, and was soon abandoned. There now came on a degree of fever, the pulse being accelerated to 100 and upwards, and soon amounting to 120. A purulent expectoration also supervened, which was discharged by a cough, very different from the original croupy cough; the respiration also being more free, as if the stricture had been partially removed.

A very respectable surgeon, Mr. Bramton of Doncaster, was sent by some friends of the patient to assist in consultation. He apprehended the case immediately by my description, before he saw the Col., and differed only in his opinion that the seat of the complaint was in the glottis. He conceived (to use his own expression) that nature had taken the alarm, and that the effort now making would be critical, but, probably, from the increasing debility, fatally so. Indeed, the day after this interview, the symptoms became so much aggravated, that I despaired of my patient surviving the day, the respiration having become so quick and laborious, the pulse pulse beating from 120 to 130, weak, and fluttering, with frequent intermissions, and the expectoration being suspended. He not only, however, survived the night, but was much better the day following; expectoration was restored, and the pulse became slower, more regular, and free. The day after he relapsed, and again was despaired of. He again rallied, and felt equal to washing himself, and changing his linen; but the exertion was carried too far; he became gradually weaker, and, though he survived four or five days, was evidently sinking. He had not lain in bed for nearly a fortnight, as he found himself easier on a sofa, being well supported with pillows. In addition to his general sufferings, he experienced much pain about this period from the hæmorrhoids, and had almost expired in consequence of changing his position to the right side, to have some suitable application put to them. Dr. Walker saw him again in this state, and recommended wine and nutritious diet, giving it as his opinion that he might continue a week or two, but would probable die suddenly. The day after, the latter part of the prediction was verified. He felt two or three convulsive arrests of breathing, and suddenly expired.

Fifteen hours after death, the body was inspected. The liver was first examined, and, to our surprise, was found, in all respects, perfectly natural. The spleen was somewhat enlarged, and almost putrid, being of a bluish white colour. The lungs were ash-coloured; livid in patches; adherent, on the left side, to the pleura costalis, in which cavity there were found about five or six ounces of a serous fluid tinged with blood. A somewhat greater quantity of a similar fluid appeared in the right cavity, but without adhesion of the lungs. The internal substance of these organs was infarcted with an effusion of bloody serum, mixed with aerated pus. The air-cells appeared, in many places, to have been ruptured, forming large projecting vesicles upon the surface of the lungs, and particularly at their edges. The pericardium contained about two ounces of colourless serum. The heart appeared rather flaccid, less florid than natural, and had, in each ventricle, a little black coagulated blood. The aorta, immediately after having emerged from the left ventricle, was dilated into a large unequal cylindrical tube, the dilatation being continued to the commencement of the arch, at which place, just before the arteria inominata is given off, an aneurismal sac, about the size of a pullet's egg, was discovered, replete with laminated coagulum of blood, and resting on the dextro-posterior part of the trachea; in the side of which, a hole about the size of a sixpence was formed, by the continued pressure of the sac.

The

The membranous covering of the sac, where it rested on the trachea, was so thin as to make it doubtful if it also were not corroded, the mass of coagulum serving as a plug both to the artery and the trachea. The dilated aorta was choked up with polypi of coagulated blood. The larynx and trachea, except where the ulceration was, were internally sound and healthy.

One of the most peculiar circumstances of this case is, the little derangement of the pulse; though, from the existence of aneurism, and the diseased state of the aorta ascendens, one might be prepared to expect great irregularity in the circulating system. Dr. Baillie has favoured me with the remark, that "it is not uncommon for the pulse, in aneurism of the arch of the aorta, not to intermit, but even to be tolerably regular." Another remarkable occurrence is the ulceration of the trachea, without the transmission of air into the aorta, or inundation of blood into the trachea. We were particular in ascertaining whether there was any emphysema, from the escape of air into the cellular membrane, but found no vestige of such communication, nor can it be supposed that the peculiar noise I before mentioned, as occurring in laborious respiration, depended on any deviation of the current of air from its natural channel. It arose, more probably, from a rupture of the air-cells on the margin of the lungs. There were, indeed, occasionally, streaks of blood in the expectorated mucus, after severe paroxysms of coughing; but they were merely the consequence of the violent exertion lacerating a few superficial vessels. From the fine and almost membranous state of the stratified coagulum of blood in the aneurismal sac, it might be concluded, that the aneurism must have been of long standing; but the inference is invalidated when we consider, that the tumour did not, at his death, which happened on the 30th of October, exceed one inch and a quarter in diameter, and that it had not attained any considerable magnitude till the beginning of the month of May, when, from the sudden loss of voice, and great dyspnœa, which were, at the time, but probably erroneously, ascribed to the effects of cold, it may be supposed it had acquired a rapid augmentation of bulk, as, till then, there had been no particular impediment to the functions of respiration. Whether the solidity of its contents is to be referred to the increased force of compression from its proximity to the heart, or to any coagulating power from the influence of the air acting upon the confined blood, through the attenuated parietes of the sac and membranous part of the trachea, I leave to more able theorists to determine. Rupture of the air-cells of the lungs is, I apprehend, a circumstance cumstance not frequently observed; but, in this case, it may reasonably be attributed to the violence of the expiratory effort, during the paroxysm of coughing; the air reacting upon the cells of the lungs, in consequence of the resistance to its exit through the contracted tube. The appearance of purulent matter, and the partial gangrene of the substance of the lungs, confirmed by the accelerated state of the pulse, and the effusion of bloody serum into the cavities of the pleuræ, denote previous inflammation of these organs, which supervened about a fortnight before the death of the patient, and may be accounted for on the supposition of the stimulus of pain, from over-distension of the air cells, acting on the vascular system, previously debilitated from imperfect oxygenation of the blood. "A really putrid state of the spleen," Dr. Baillie informs me, "is very rare; but an extremely soft state of it, without putridity, is not uncommon." In this case it had more the appearance of a mass of grumous blood, scarcely confined by its very tender covering, and was not of sufficient tenacity to bear displacing by the hand, without the fingers passing through its substance. It is very singular that there was no suspicion of its being at all diseased; and it is equally so that the liver should present itself in a healthy state, having been so generally suspected of derangement of structure.

*Pontefract;* JOHN HAXBY, M.D.
*Feb.* 24, 1815.

*Remarks of an Editor.*—The above case is in all respects so valuable, and related with so much perspicuity and candour, that we cannot refrain from expressing thanks for our readers as well as ourselves. We trust to Dr. Haxby's candour that he will not be displeased with a few inquiries. First, might not the cause of the dyspnœa be the occasional pressure of the tumour on the upper part of the œsophagus? Secondly, was any attempt made to press the air from the larger vesicles into the cells of the lungs? Such vesicles seem to owe their origin to the rupture of a number of cells by some such violence as Dr. Haxby relates. We are informed by a learned veterinary surgeon, that they are the usual cause of the broken-wind in horses. From the violence which occasions the rupture, probably inflammation follows, by which adhesions take place, thus effectually separating the ruptured from the sound air-cells. If such was not the case in the present instance, it renders the appearance still more interesting, from its rarity, and the effect produced during life. Lastly, might not the appearances in the spleen, and of gangrene in the lungs, be the effect of unoxygenated blood loosely coagulated, as it was found in the heart?

