# Errata

**Published:** 1815-06

**Authors:** 


I shall be obliged to you to notice the following errata in No. 194.

P. 266, line 30, instead of "*from home,*" read "*from this place,*"

P. 268, line 11, for "*Spirit of Æther,*" read "*Spirit of Nitrous Æther.*"

P. 269, line 37, for "*Mr. Bramton,*" read "*Mr. Branston.*"

P. 271, line 29, for "*the fine,*" read "*the firm.*"

